# Efficient selective hydrogenation of *N*,*N*-dimethylaniline in a continuous fixed-bed reactor over a Cu/Ni–Al_2_O_3_ catalyst

**DOI:** 10.1039/d6ra01119e

**Published:** 2026-04-13

**Authors:** Jingdong Nong, Zhonghua Sun, Weiyou Zhou, Zhong Wu, Junfeng Qian, Qun Chen, Mingyang He

**Affiliations:** a Jiangsu Key Laboratory of Advanced Catalytic Materials and Technology, Changzhou University 213164 Changzhou China sunzhonghua@cczu.edu.cn

## Abstract

A series of Cu-modified Ni–Al_2_O_3_ catalysts with various Cu/Ni ratios (*x*Cu/Ni–Al_2_O_3_) were prepared, and systematically characterized using BET, XRD, SEM, H_2_-TPR, H_2_-TPD, TG, and XPS. The catalyst exhibited excellent catalytic performance in the selective hydrogenation of *N*,*N*-dimethylaniline to *N*,*N*-dimethylcyclohexylamine in a continuous fixed-bed reactor. When the Cu/Ni molar ratio was 0.1, the active components were highly dispersed on the Al_2_O_3_ support, which possessed a high specific surface area and well-developed porous structure. Furthermore, the catalyst demonstrated strong hydrogen desorption capacity and enhanced thermal stability. Synergistic electronic interactions between Ni and Cu improved the adsorption and activation of both reactant and H_2_ molecules. The incorporation of Cu near the Ni active sites effectively modulated the selectivity toward the desired product *N*,*N*-dimethylcyclohexylamine. Under the optimal reaction conditions: H_2_ pressure of 5 MPa, reaction temperature of 130 °C, liquid hourly space velocity 0.1 h^−1^ and hydrogen-amine volume ratio of 5500 : 1, the 0.1Cu/Ni–Al_2_O_3_ catalyst achieved superior performance, with a *N*,*N*-dimethylaniline conversion rate of 99.5% and a selectivity for *N*,*N*-dimethylcyclohexylamine of 97.6%.

## Abstract 2

An efficient catalytic system based on a copper-doped nickel-alumina catalyst (*x*Cu/Ni–Al_2_O_3_) has been developed for the selective hydrogenation of *N*,*N*-dimethylaniline (*N*,*N*-DMA) to *N*,*N* dimethylcyclohexylamine in a continuous fixed-bed reactor.

## Graphical abstract

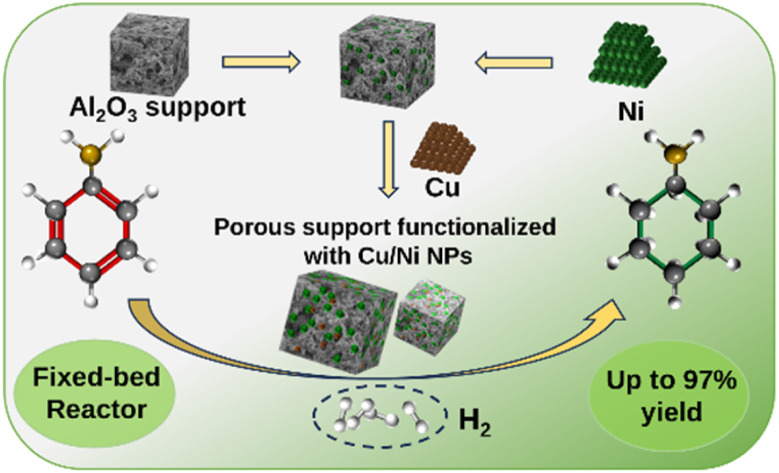

## 1 Introduction

Amines, as a class of important industrial organic compounds, play an indispensable role in the production of pharmaceutical intermediates, resin precursors, rubber stabilizers, corrosion inhibitors, and plastics.^1^ Among these, *N*,*N*-dimethylcyclohexylamine (DMCHA), a significant fine chemical within the tertiary amine family, functions as an efficient industrial catalyst in the processing and molding of rigid polyurethane (RPUR) plastics.^2,3^ Furthermore, DMCHA exhibits tunable hydrophilicity, making it a commercially available and irreplaceable switchable hydrophilic solvent (SHS).^4^ These attributes underscore the promising market prospects of DMCHA, whose diverse application domains and unique physicochemical properties establish it as a critical raw material across multiple industries.

Traditionally, DMCHA is synthesized *via* the reductive amination of cyclohexanone with dimethylamine. However, this method suffers from several inherent limitations, including low selectivity, susceptibility to side reactions, dehalogenation or over-reduction,^5–7^ Recently, Yu *et al.*^8^ developed a supported Pd/C catalyst that achieved relatively high yields of DMCHA in a continuous-flow micro-packed bed reactor system. While this approach mitigates some drawbacks of conventional reductive amination, the high cost of the Pd/C catalyst poses economic and sustainability challenges, restricting its potential for large-scale industrial application. The palladium and ruthenium catalysts supported on alumina, investigated by Palkovics *et al.*,^9^ effectively catalyzed the hydrogenation of ethyl anisate and dimethyl anisate in a continuous flow system, achieving a 90% yield of DMCHA. Similarly, the raw materials for its catalyst are highly costly, which hinders large-scale production, and the reaction yield remains relatively low. Therefore, compared to the conventional reductive amination approach, the synthesis of cyclohexylamine and its derivatives *via* hydrogenation of aromatic amines offers distinct advantages in terms of synthetic steps and cost efficiency.

The one-step hydrogenation of *N*,*N*-dimethylaniline (*N*,*N*-DMA) for DMCHA synthesis is characterized by its straightforward pathway, environmental compatibility, and high efficiency.^10–13^ In previous studies, various catalysts, including noble metals (Rh, Ru, Pd) and non-noble metals (Fe, Co, Ni, Cu), have been employed for the hydrogenation of aniline and its derivatives.^10–13^ The hydrogenation of aromatic amines to alicyclic amines represents a well-established and strategically important transformation in organic synthesis.^14,15^ Considerable efforts have been devoted to achieving complete benzene ring hydrogenation in aromatic amines. For example, J. Wu *et al.* developed a Pt–Ru/C_3_N_4_ bimetallic catalyst capable of accomplishing tandem nitro and benzene ring hydrogenation under base-free conditions, elucidating the synergistic mechanism of bimetallic co-hydrogenation.^16^ X. Lu *et al.* utilized nickel-based catalysts doped with 3‰ Rh for the hydrogenation of nitrobenzene, achieving 100% conversion and 91.6% selectivity toward cyclohexylamine.^10^ Currently, research on the hydrogenation synthesis of DMCHA from *N*,*N*-DMA has primarily been conducted through batch-type reactor experiments. For example, Mao *et al.*^17^ developed a NiRe/HAP catalyst by incorporating Re as a promoter, which significantly enhanced the hydrogenation activity of Ni. This catalytic system demonstrated high efficiency in converting aromatic and heteroaromatic compounds, achieving a DMCHA yield of up to 99%. Nevertheless, this approach suffers from limited production capacity, low separation efficiency, and reliance on Re, thus posing challenges for large-scale industrial application. Similarly, Murugesan and Kuma *et al.*^18,19^ reported nano-cobalt and nano-oxidized Ru catalysts, respectively, both of which exhibited efficient and selective hydrogenation of aromatic hydrocarbons, yielding DMCHA at 90% and 94%. However, these powdered catalysts suffer from poor recyclability and separation. Therefore, developing efficient catalysts for selective *N*,*N*-DMA hydrogenation in a continuous-flow process is highly desirable for industrial applications.

In this study, a series of Cu-modified Ni–Al_2_O_3_ catalysts with various Cu/Ni ratios (*x*Cu/Ni–Al_2_O_3_) were prepared, systematically characterized, and investigated in the selective hydrogenation of *N*,*N*-DMA towards DMCHA in a continuous fixed-bed reactor. The effect of different metal promoters (Fe, Co, Ru, Cu, Sn) on catalytic performance was systematically investigated.^20–23^ Cu was identified as the most effective promoter, and synergistic effect was observed between them. Subsequently, the effect of varying Cu/Ni molar ratios on the catalytic hydrogenation of *N*,*N*-DMA was investigated. Furthermore, key reaction parameters-including hydrogen pressure, reaction temperature, liquid hourly space velocity (LHSV) and hydrogen-amine volume ratio were optimized to maximize catalytic performance. Under the refined conditions, the 0.1Cu/Ni–Al_2_O_3_ catalyst exhibited superior activity, achieving high substrate conversion while significantly enhancing DMCHA selectivity. Notably, the catalyst demonstrates excellent mechanical strength and rapid mass transfer properties, making it highly suitable for industrial-scale implementation.

## 2 Experimental

### 2.1 Materials and chemicals

*N*,*N*-dimethylaniline (C_8_H_11_N, AR) and methanol (CH_3_OH, AR) were purchased from Shanghai Aladdin Biochemical Technology Co., Ltd. RuCl_3_·*x*H_2_O (Ru: 37.0wt%), SnCl_4_ (AR), CoN_2_O_6_·6H_2_O (AR), FeN_3_O_9_·6H_2_O (AR) and CuN_2_O_6_·6H_2_O (AR), NiN_2_O_6_·6H_2_O and Al_2_O_3_ (99.9% purity) were purchased from Sinopharm Chemical Reagent Co., Ltd (Shanghai, China). All reagents were of analytical reagent grade and used without further treatment. High purity hydrogen (>99.9%) and high purity nitrogen (>99.9%) were purchased from Jiang Su Shenlian Industrial Gas Co., Ltd.

### 2.2 Preparation of catalyst

#### 2.2.1 Preparation of Ni–Al_2_O_3_ precursor

The bimetallic catalyst precursor with a Ni mass fraction of 30%, designated as *x*/Ni–Al_2_O_3_, was synthesized *via* the excess impregnation method. Initially, Ni(NO_3_)_2_·6H_2_O was heated to 80 °C in an oil-bath pan until fully dissolved. Subsequently, the Al_2_O_3_ support was introduced into the solution and fully immersed, followed by impregnation for 2 h with stirring at 30 min intervals. Following impregnation, the Ni(NO_3_)_2_·6H_2_O solution was recovered and can be reused, and residual solution on the surface of the Ni–Al_2_O_3_ was removed by washing, prevent the remaining Ni(NO_3_)_2_·6H_2_O liquid from condensing upon cooling, which could cause the catalyst to clog. After impregnation, the Ni–Al_2_O_3_ was subjected to vacuum filtration and aged at room temperature for 8 hours. The sample was then dried in an oven at 100 °C for 12 h and calcined at 350 °C for 3 h with a heating rate of 2 °C min^−1^. To achieve a nominal Ni loading of 30 wt%, this sequence of impregnation, drying and calcination was repeated three times to obtain the final bimetallic catalyst precursor labeled *x*/Ni–Al_2_O_3._

#### 2.2.2 Preparation of *x*Cu/Ni–Al_2_O_3_ catalyst

The Ni–Al_2_O_3_ precursor was added to an aqueous copper nitrate solution, which was prepared by dissolving a specified amount of Cu(NO_3_)_2_·3H_2_O in deionized water. The mixtures were then evaporated at 80 °C, then the sample was dried in an oven at 100 °C for 10 h and calcined in air at 400 °C for 4 h with a heating rate of 2 °C min^−1^, the calcined sample labeled as *x*Cu/Ni–Al_2_O_3_. The *x*Cu/Ni–Al_2_O_3_ were reduced by H_2_ at 450 °C for 3 h to obtain *x*Cu/Ni–Al_2_O_3_ catalysts with the Cu content of 0.05 wt%, 0.1 wt%,0.3 wt%, and 0.5 wt%, which were labeled as 0.05Cu/Ni–Al_2_O_3_, 0.1Cu/Ni–Al_2_O_3_, 0.3Cu/Ni–Al_2_O_3_, and 0.5Cu/Ni–Al_2_O_3_, respectively.

### 2.3 Catalyst characterization

#### 2.3.1 Nitrogen adsorption–desorption analysis

The Autosorb-iQ2-MP physical adsorption instrument (Quantachrome Instruments, United States) was used to measure the N_2_ adsorption–desorption isotherms and pore size distributions of the xCu/Ni–Al_2_O_3_ catalyst. First, 80 mg of the sample was pretreated under vacuum at 110 °C for 3 h. Subsequently, the N_2_ adsorption–desorption isotherms of the sample were tested at −196 °C in a liquid nitrogen atmosphere. The specific surface area of the catalyst was calculated using the BET equation, and the pore volume and pore size were determined from the desorption branch of the isotherm *via* the BJH method.

#### 2.3.2 XRD characterization

Powder X-ray diffraction (XRD) measurements were performed using a D/max2500 PC X-ray diffractometer (Rigaku, Japan) to analyze the crystal structure of the powder sample. The X-ray source (Cu kα1,2, *λ* = 0.15418 Å) operated at 40 kV and 100 mA. The diffraction angle 2*θ* ranged from 5° to 80° with a scanning rate of 12 (°) min^−1^, The collected diffraction data were analyzed and processed by software (MDI Jade 9) and PDF-4+ database.

#### 2.3.3 SEM analysis

The morphology and structure of the *x*Cu/Ni–Al_2_O_3_ catalyst were analyzed using the German-Zeiss Sigma 360 field emission scanning electron microscope (FE-SEM, Zeiss, Germany). The test conditions were as follows: electron microscope resolution of 5.0 nm, acceleration voltage of 5.0 kV, aperture size of 30.0 µm, and magnification of 1000×. For the sample preparation. After 5 minutes of ultrasonic treatment, the catalyst sample was uniformly dispersed, and the sample (metal powder) was directly adhered to a conductive adhesive. After drying, the sample was placed under the microscope for imaging.

#### 2.3.4 H_2_-TPR studies

The catalyst was analyzed by AutoChem Ⅱ chemical adsorption analyzer (Micromeritics, USA). The test conditions were as follows: 80 mg of the *x*Cu/Ni–Al_2_O_3_ catalyst sample was taken and pretreated at 200 °C for 1 hour under Ar atmosphere. Next, the samples was cooled to room temperature, purged with H_2_/Ar mixed gas, and then heated from room temperature to 800 °C at a heating rate of 10 °C min^−1^ for desorption. The desorbed gas was detected by a thermal conductivity detector (TCD), and the H_2_-TPR profiles were obtained.

#### 2.3.5 H_2_-TPD studies

The *x*Cu/Ni–Al_2_O_3_ catalyst was analyzed by AutoChem Ⅱ chemical adsorption instrument of MAC Instrument Corporation. The test conditions were as follows: 80 mg of the sample was loaded into the U-type quartz tube reactor and pretreated at 350 °C for 1 hour under H_2_ (50 mL min^−1^) atmosphere, then cooled to room temperature, purged with Ar mixed gas (20 mL min^−1^), purged back to Ar to remove physically adsorbed H_2_. and then the sample was desorbed from room temperature to 700 °C at a ramp rate of 10 °C min^−1^, finally, the H_2_-TPD profiles were obtained.

#### 2.3.6 TG analysis

Thermogravimetric analysis (TG) was performed on a TG-209-F3 thermogravimetric analyzer. A 3 mg sample was placed in an alumina crucible and heated from 40 °C to 800 °C at a rate of 15 °C min^−1^ under 99.99% N_2_ flow (15 mL min^−1^), finally, the TG profiles were obtained.

#### 2.3.7 X-ray photoelectron spectroscopy XPS

X-ray photoelectron spectroscopy (XPS) analysis was performed using monochromatic Al Kα radiation (*hv* = 1486.6 eV) as the excitation source. Catalyst samples were prepared by pressing powder into a pellet and then transferred into the sample chamber of a Thermo Scientific K-Alpha XPS instrument. Once the pressure in the sample chamber dropped below 2.0 × 10^−7^ mbar, the sample was transferred to the analysis chamber under vacuum conditions. High-resolution narrow scans were conducted with a pass energy of 50 eV and a step size of 0.1 eV. The Ni2p and Cu2p binding energies (BEs) were recorded. Charge correction was applied by referencing the C1s peak to 284.80 eV. All data were analyzed using a software (avantage).

### 2.4 Evaluation of catalytic performance

The catalytic performance of the *x*Cu/Ni–Al_2_O_3_ catalyst for *N*,*N*-DMA hydrogenation was evaluated in a high-pressure fixed-bed reactor. 20 mL of the catalyst sample was filled into the middle section of a three-stage stainless steel reaction tube, with quartz sand filled in the upper and lower ends for fixation. A thermocouple was inserted from the bottom of the stainless-steel tube. The thermosensitive element to extend to the core area of the catalyst bed to monitor the temperature changes during the hydrogenation reaction in real time.

**Scheme 1:**
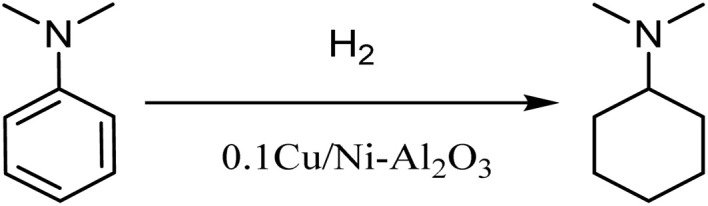
*N*,*N*-DMA hydrogenation reaction.

Pressurized hydrogen was flowed downward through the reactor containing the catalyst bed, using an electronic mass flow controller to control the hydrogen gas flow rate. The temperature of the fixed bed was programmed using a temperature control system. Typically, the heating rate of the fixed-bed reactor was set at 10 °C min^−1^. Since the reaction was performed under high pressure, *N*,*N*-DMA was fed using a reciprocating pump, and liquid product samples were collected at regular intervals (every 2 h). The reaction scheme for the hydrogenation of *N*,*N*-DMA to DMCHA is illustrated in Scheme 1.

The obtained liquid samples were monitored using a gas chromatography (GC, Shimadzu GCMS-QP2010) equipped with a flame ionization detector (FID). The temperature of FID detector was set at 280 °C. An HP-5 capillary column (30 m × 0.32 mm × 1.00 µm) was used with nitrogen as the carrier gas at a flow rate of 1 mL min^−1^. The injection volume was 0.2 µL with a split ratio of 10 : 1. Maintain the sample port temperature at 240 °C and the column oven temperature at 100 °C for 2 minutes. The temperature was raised to 240 °C at a rate of 20 °C min^−1^ and maintained for 10 minutes. The conversion of *N*,*N*-DMA (C) and the selectivity of DMCHA (S) were calculated using the following equations:
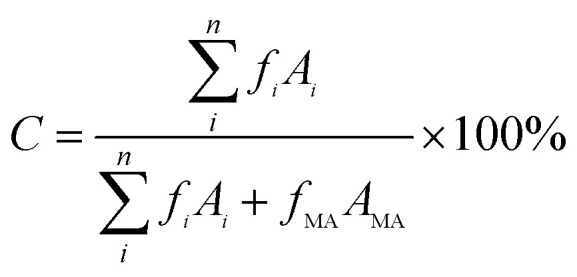

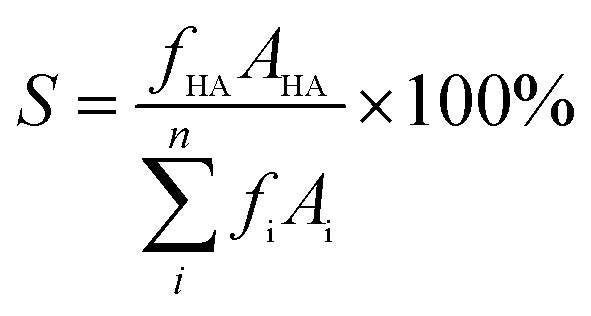
where *A*_MA_, *A*_HA_, and *A*_i_ are the peak areas of *N*,*N*-DMA, DMCHA, and each product, respectively. *f*_MA_, *f*_HA_, and *f*_i_ are the correction factor of *N*,*N*-DMA, DMCHA, and each product, respectively.

## 3 Results and discussion

### 3.1 Synthesis and characterization of *x*Cu/Ni–Al_2_O_3_ catalysts

#### 3.1.1 Nitrogen adsorption–desorption analysis

**Fig. 1:**
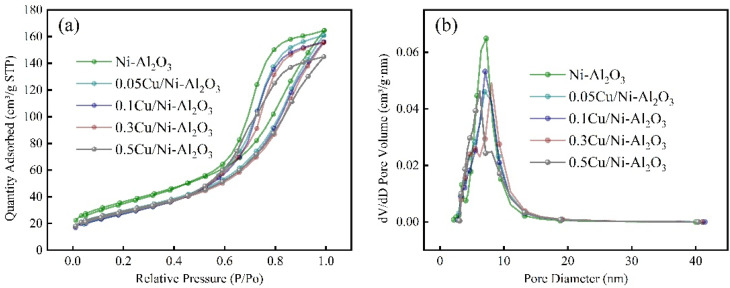
(a) N_2_ adsorption–desorption isotherms and (b) pore size distribution of the *x*Cu/Ni–Al_2_O_3_ catalysts and Ni–Al_2_O_3_ precursor.

The nitrogen adsorption–desorption isotherms and pore size distribution curves of the synthesized *x*Cu/Ni–Al_2_O_3_ catalysts and the Ni–Al_2_O_3_ precursor are presented in Fig. 1. According to the IUPAC classification, the *x*Cu/Ni–Al_2_O_3_ catalysts exhibit typical type IV isotherms with H1 hysteresis loops across varying *P*/*P*_0_ values, indicative of well-defined mesoporous structures. In the low-pressure region, nitrogen adsorption occurs primarily through monolayer formation within the pore network, followed by a sharp increase in capillary condensation in the medium-pressure range, which contributes to the development of hysteresis loops.^24^ A distinct H1 hysteresis loop is observed in the relative pressure range of *P*/*P*_0_ = 0.55–1.0, confirming the presence of cylindrical or uniform mesopores. These findings suggest that the prepared *x*Cu/Ni–Al_2_O_3_ catalysts possess characteristic mesoporous features, indicating that such materials typically exhibit narrow pore size distributions and interconnected porous channels.^25^ The resulting high specific surface area and pore volume facilitate the highly dispersed distribution of active nickel and copper species on both the surface and internal pore walls of the catalyst, as corroborated by SEM observations. Furthermore, the well-ordered mesoporous architecture (pore diameters ranging from 2 to 50 nm) reduces mass transfer resistance, thereby enhancing the accessibility of reactant molecules to active sites. This structural advantage promotes efficient interaction between catalytic centers and reactants, ultimately improving hydrogenation catalytic performance.

As shown in Fig. 1, with increasing copper loading, both the N_2_ adsorption–desorption isotherms and pore size distribution curves decrease, reflecting a reduction in specific surface area and pore volume. This trend can be attributed to the partial blockage and occupation of alumina support pores by an increased amount of copper species. This variation in the specific surface area and pore structure of the catalyst is evident from Table 1. Notably, When the Cu/Ni molar ratio is 0.1, the catalyst exhibits the smallest specific surface area and pore volume, indicating that the active species influence the pore structure as well as the surface filling and coverage of the support. Meanwhile, the appropriate incorporation of Cu reduces the continuity of Ni active sites, thereby exerting minimal impact on the main reaction while significantly suppressing the side reaction.

**Table 1:** BET and BJH analysis of catalysts with different Cu/Ni molar ratios

Catalyst	Specific surface area (m^2^ g^−1^)	Average pore diameter (nm)	Total pore volume (mL g^−1^)
Ni–Al_2_O_3_	129.1	6.9	0.25
0.05Cu/Ni–Al_2_O_3_	105.7	7.0	0.24
0.1Cu/Ni–Al_2_O_3_	101.6	7.1	0.22
0.3Cu/Ni–Al_2_O_3_	102.3	7.3	0.23
0.5Cu/Ni–Al_2_O_3_	104.3	6.7	0.24

#### 3.1.2 XRD characterization

**Fig. 2:**
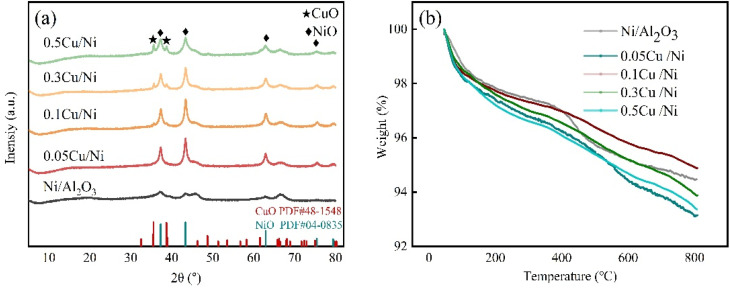
(a) XRD patterns of Ni–Al_2_O_3_ and *x*Cu/Ni–Al_2_O_3_ precursor. (b) TG profiles of Ni–Al_2_O_3_ and *x*Cu/Ni–Al_2_O_3_ precursor.

Fig. 2(a) shows the XRD patterns of *x*Cu/Ni–Al_2_O_3_ catalysts with different Cu/Ni molar ratios and Ni–Al_2_O_3_ precursors. As shown in the figure, the peaks at 2*θ* = 37.3°, 43.4°, 62.9° and 75.5° correspond to the cubic crystal structure of NiO (JCPDS: 04-0835), and the peaks at 2*θ* = 35.6°, 38.7° and 48.9° correspond to the cubic crystal structure of CuO (JCPDS: 48-1548) in the analysis of the crystalline phase of the catalyst and the precursor. The characteristic XRD diffraction peak intensity of NiO in the Ni–Al_2_O_3_ precursor is weak, indicating that NiO is highly dispersed on the surface of the Al_2_O_3_ carrier. This is mainly because the carrier has a large specific surface area and excellent pore structure, so that the unreduced active components NiO and CuO can be uniformly dispersed on the surface of the carrier.^26^

The XRD diffraction peak intensity gradually increased as the introduction of Cu, and the half-peak width of NiO became narrower, revealing that the crystallinity and crystal size of NiO crystals increased correspondingly, mainly because the addition of CuO induced lattice distortion.^27^ With the increase of the Cu/Ni ratio, the diffraction peak intensity of CuO increased correspondingly because more CuO lattices accumulated on the surface of the catalyst, and excess CuO would cover the position of NiO on the carrier. At the same time, the half-peak width of NiO diffraction peak gradually widened and the intensity decreased, which would lead to the reduction of the contact between Ni^2+^ and H^+^,^28^ excessive inhibition of hydrogenation performance, and reduction of reactant conversion. Therefore, the improved catalytic performance should be related to the appropriate Cu/Ni ratio.

#### 3.1.3 TG analysis

Fig. 2(b) shows the TG analysis results of *x*Cu/Ni–Al_2_O_3_ and Ni–Al_2_O_3_ precursor. The initial stage of weight loss occurs within the temperature range of 50–200 °C, during which the TG curve exhibits a sharp decline. This weight loss is primarily attributed to the removal of physically adsorbed water.^29^ Although the catalyst was calcined at 350 °C prior to analysis, it reabsorbed moisture from the ambient environment before the TG measurement, indicating that the calcined catalyst possesses a well-developed pore structure, a characteristic feature of Al_2_O_3_ support materials. In the temperature interval of 200–450 °C, the TG curve remains relatively stable, suggesting that the decomposition of nickel nitrate, introduced *via* incipient wetness impregnation, was largely completed during the calcination process. Therefore, a calcination temperature of 350 °C is sufficient and effective for the preparation of the catalyst. Consequently, the precursor of the active phase has already been converted into the desired oxide form, which is consistent with the XRD characterization results. Beyond 450 °C, the curve undergoes another pronounced weight loss, which may be associated with the decomposition of residual intermediate species-such as basic nickel nitrates formed during the initial calcination step at 350 °C and requiring higher temperatures for complete decomposition.^30^

#### 3.1.4 H_2_-TPR studies

**Fig. 3:**
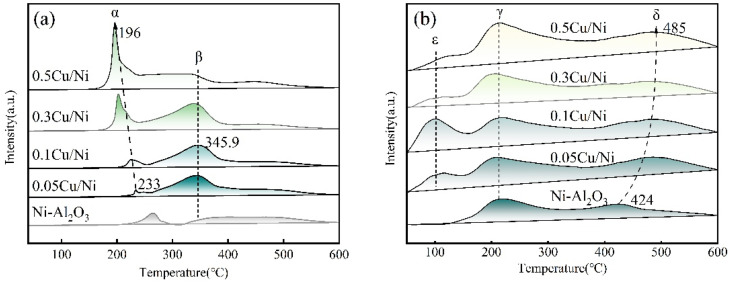
(a) H_2_-TPR profiles of *x*Cu/Ni–Al_2_O_3_ and Ni–Al_2_O_3_ precursor samples. (b) H_2_-TPD profiles of *x*Cu/Ni–Al_2_O_3_ and Ni–Al_2_O_3_ precursor samples.

The reduction behaviors of *x*Cu/Ni–Al_2_O_3_ and Ni–Al_2_O_3_ precursor samples were investigated *via* H_2_-TPR analysis. Fig. 3(a) presents the H_2_-TPR profiles of the 0.05Cu/Ni–Al_2_O_3_, 0.1Cu/Ni–Al_2_O_3_, 0.3Cu/Ni–Al_2_O_3_, 0.5Cu/Ni–Al_2_O_3_ and Ni–Al_2_O_3_ precursors. The Ni–Al_2_O_3_ precursor sample exhibits a prominent H_2_ consumption peak within the temperature range of 200–300 °C, with a relatively large peak area. This behavior is primarily attributed to the high specific surface area and well-developed mesoporous structure of the Ni–Al_2_O_3_ precursor, which facilitate dispersion and adhesion of NiO species on the surface and in the pores of the support. Consequently, H_2_ molecules achieve enhanced contact with NiO, promoting its efficient reduction. Upon Cu incorporation, two additional H_2_ consumption peaks emerge, designated as peak α (190–240 °C) and peak β (300–400 °C). With increasing Cu/Ni ratio, both the intensity and area of peaks α and β increase, and their corresponding peak temperatures exhibit distinct shifts across the *x*Cu/Ni–Al_2_O_3_ series. Peak α, located in the low-temperature region, is assigned to the reduction of highly dispersed NiO species to Ni^0^ and CuO species to Cu_2_O.^27^ In contrast, peak β, observed at higher temperatures, corresponds to the reduction of aggregated NiO species and Cu_2_O to Cu^0^, as well as NiO species confined within the support pores. These species are less accessible and require higher reduction temperatures due to stronger interactions with the support, indicating enhanced metal–support interaction in the bimetallic system.

With increasing Cu content, peak α progressively shifted toward lower temperatures, while its area increased, suggesting that Cu promoted the reduction of NiO species. Additionally, more surface CuO species are reduced at lower temperatures, reflecting improved H_2_ adsorption capacity and enhanced low-temperature reducibility in the CuNi bimetallic system compared to the monometallic counterpart, thereby favoring hydrogenation reactions. For the 0.05Cu/Ni–Al_2_O_3_, 0.1Cu/Ni–Al_2_O_3_, 0.3Cu/Ni–Al_2_O_3_ and 0.5Cu/Ni–Al_2_O_3_ precursors, peak β initially shifted to higher temperatures and then shifted back to lower temperatures as the Cu/Ni ratio increased. The highest reduction temperature for peak β is observed at 345.9 °C for 0.1Cu/Ni–Al_2_O_3_. This phenomenon can be attributed to the optimal Cu/Ni molar ratio facilitating the formation of structurally CuNi alloy clusters, whose stability delays reduction and necessitates higher temperatures,^31^ the strong interaction between Cu and Ni attenuates Ni's ability for dissociative adsorption of H_2_ and activation of C–N bonds, thereby exerting a more pronounced inhibitory effect on the demethylation side reaction. Furthermore, the area of peak β increases with Cu loading, owing to the greater amount of CuO deposited on the support. Compared to NiO, CuO exhibits higher reducibility under similar conditions.^32^

#### 3.1.5 H_2_-TPD studies

The hydrogen adsorption capacity and desorption energy barrier of *x*Cu/Ni–Al_2_O_3_ and Ni–Al_2_O_3_ precursors were investigated *via* H_2_-TPD experiments. The resulting H_2_-TPD profiles are presented in Fig. 3(b). For the Ni–Al_2_O_3_ precursor, two distinct H_2_ reduction peaks were observed within the temperature ranges of 150–300 °C and 380–480 °C, designated as peak γ and peak δ, respectively. Peak γ, located in the low-temperature region, is primarily attributed to the presence of NiO species dispersed on the surface of the support, which are highly accessible to H_2_ molecules and thus exhibit lower hydrogen adsorption strength. In contrast, peak δ appears at higher temperatures due to the more energetically demanding reduction of NiO species or aggregates confined within the support channels, requiring elevated temperatures for effective hydrogen activation and stronger hydrogen adsorption.^33^ The high dispersion of active components is facilitated by the mesoporous structure of the support, a finding consistent with BET characterization results.

Upon incorporation of Cu into the Ni–Al_2_O_3_ precursor, an additional H_2_ desorption peak (designated as peak ε) emerged in the H_2_-TPD profile of *x*Cu/Ni–Al_2_O_3_. With increasing Cu/Ni molar ratio, peak ε broadens progressively, suggesting that Cu enhances the dispersion of Ni species. Concurrently, peak γ shifts toward lower temperatures while peak δ shifts toward higher temperatures, indicating the influence of electronic interactions in the CuNi bimetallic system on the catalyst's hydrogen adsorption behavior. The appearance of three distinct reduction peaks in the *x*Cu/Ni–Al_2_O_3_ precursor reflects heterogeneous hydrogen adsorption sites arising from intermetallic electron interactions,^34,35^ When the Cu/Ni molar ratio is 0.1, the ε area of the low-temperature H_2_ desorption peak is the largest, indicating that the number of weakly chemically adsorbed hydrogen species on the catalyst surface is the highest. This is mainly attributed to the strong bimetallic synergistic interaction between Cu and Ni, resulting in the formation of a Cu–Ni alloy phase, continuous and regular crystal plane spacing stripes can be seen in the HR-TEM images (Fig. S3), no individual lattice stripes of Cu (*d* = 0.208 nm, 111 crystal plane) or Ni (*d* = 0.203 nm, 111 crystal plane) were observed.^36,37^ Instead, characteristic lattice stripes (*d* = 0.205 nm) that were between the interplanar spacings of the Cu and Ni crystal planes were present, indicating that Cu atoms had successfully been incorporated into the Ni lattice, forming a Cu–Ni substitutional alloy phase. This phenomenon is confirmed by SEM elemental mapping and XPS electron transfer analysis This ensures the hydrogenation of the aromatic rings in the main reaction. The weakly adsorbed hydrogen activity is insufficient to attack the strong C–N bonds, thereby inhibiting the formation of by-products and improving the selectivity.

#### 3.1.6 SEM analysis

**Fig. 4:**
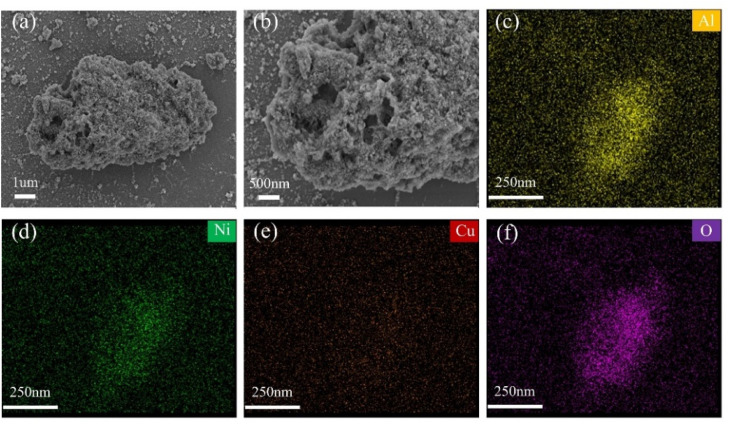
(a and b) SEM images and (c–f) elemental mappings of 0.1Cu/Ni–Al_2_O_3_ catalyst.

The morphological and structural characteristics of the 0.1Cu/Ni–Al_2_O_3_ catalyst were investigated using scanning electron microscopy, with the results presented in Fig. 4. As shown in Fig. 4(a and b), the catalyst exhibits a pronounced agglomeration phenomenon, with the sample predominantly existing in an aggregated form that constitutes large, block-like structures. The surface morphology is rough and non-uniform, with numerous fine particles randomly distributed and no distinct ordered arrangement. Additionally, well-developed porous structures are observed within the aggregates, which can be attributed to the inherent textural properties of the Al_2_O_3_ support material known for its high specific surface area. The formation of a hybrid morphology comprising both aggregates and fine particles is primarily due to the impregnation synthesis method: differences in reaction kinetics may lead to the initial formation of Cu-based nuclei, followed by the subsequent deposition of Ni species.^34^ The larger aggregates, benefiting from an increased surface area, enhance reactant adsorption and provide a greater number of accessible metal active sites, thereby contributing to improved catalytic efficiency. Furthermore, the fine particles dispersed on the aggregate surfaces serve as primary hosts for active sites, where Cu and Ni exist in the form of CuNi alloy or CuO/NiO phases-consistent with the XRD and XPS analyses. As illustrated in Fig. 4(d and e), elemental mapping confirms the uniform distribution and high dispersion of Ni and Cu across the Al_2_O_3_ support, indicating effective mixing of the metallic components. Moreover, SEM-EDS analysis (Table S1) shows that the Cu/Ni molar ratio in the 0.1Cu/Ni–Al_2_O_3_ catalyst is close agreement with the theoretical values. This consistency confirms the precision of the metal loading procedure during catalyst synthesis and demonstrates homogeneous elemental distribution, with no statistically significant deviations observed. In the elemental mappings, these surface-localized fine particles function as bimetallic active sites, with coexisting Cu and Ni species. The Cu–Ni alloy phase significantly enhances catalytic activity and stability, exhibiting a synergistic effect in hydrogenation reactions.^38^

#### 3.1.7 X-ray photoelectron spectroscopy analysis

**Fig. 5:**
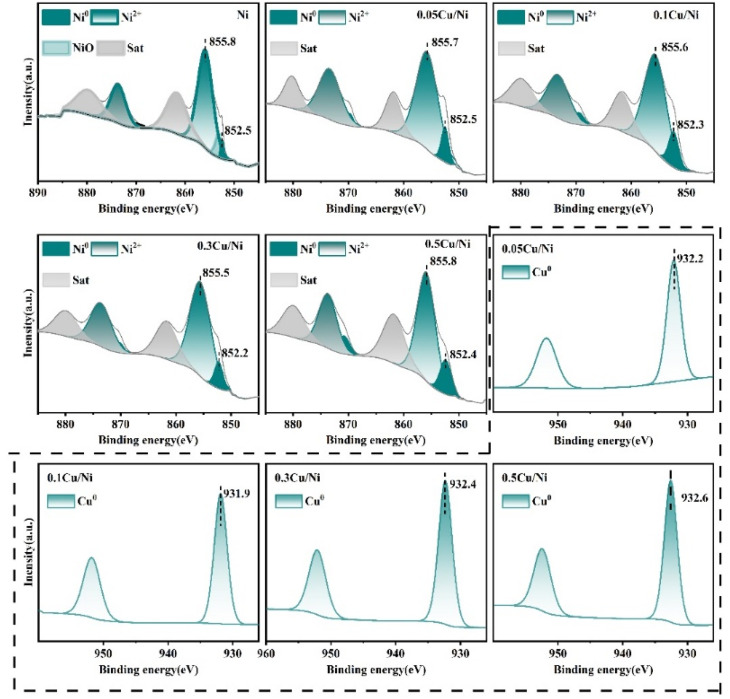
*In situ* XPS spectra of Ni 2p for the Ni–Al_2_O_3_, *x*Cu/Ni–Al_2_O_3_ catalyst (outside the dashed area) and Cu2p for the *x*Cu/Ni–Al_2_O_3_ catalyst (within the dashed line area).

The surface elemental composition and chemical states of the metals in the *x*Cu/Ni–Al_2_O_3_ catalyst were investigated using X-ray photoelectron spectroscopy. As illustrated in Fig. 5 (outside the dashed area), the Ni 2p_3/2_ spectrum exhibits characteristic peaks corresponding to Ni^0^ (852.2–852.5 eV), NiO (853.2 eV) and Ni(OH)_2_ (855.5–855.8 eV), along with satellite peaks arising from the strong interaction between Ni^2+^ species and the Al_2_O_3_ support. This indicates significant metal–support interaction within the *x*Cu/Ni–Al_2_O_3_ system. With increasing copper content (Cu/Ni molar ratios of 0.05, 0.1 and 0.3), the binding energies of Ni^0^ (852.5 eV) and Ni(OH)_2_ (855.8 eV) shift toward lower values, suggesting electron transfer from Cu to Ni. However, at higher copper loading (Cu/Ni = 0.5), the binding energies of these species increase again, indicating bidirectional electron interaction: Ni facilitates Cu reduction while Cu donates electron density to Ni, thereby demonstrating strong electronic coupling between the two metals.^17^ These findings suggest that an optimal Cu/Ni molar ratio enhances catalytic activity.

The Cu 2p XPS spectrum displays distinct spin–orbit splitting, as shown in Fig. 5 (within the dashed line area). The Cu 2p_3/2_ peak for Cu^0^ is observed at 931.9–932.6 eV, with the corresponding Cu 2p_1/2_ peak appearing at 951.8–952.5 eV. When the Cu/Ni molar ratio increases from 0.05 to 0.1, a slight decrease in the Cu 2p_3/2_ binding energy is observed, reflecting the electronic interaction between Ni and Cu. Metallic Ni has a higher density of d-band holes than Cu, promoting the transfer of d electrons from Cu^0^ to vacant d orbitals of Ni^0^ and resulting in electron-deficient Cu species.^39^ This synergistic electronic effect facilitates the formation of well-dispersed and highly adsorptive bimetallic Cu–Ni species on the Al_2_O_3_ support, thereby enhancing the catalytic performance of the 0.1Cu/Ni–Al_2_O_3_ catalyst. However, at higher copper loadings (Cu/Ni = 0.3 and 0.5), excessive copper leads to surface enrichment of Cu species, which may mask or block certain Ni active sites, disrupting the balance between the two metals and ultimately reducing overall catalytic efficiency.

### 3.2 Catalytic activity test

#### 3.2.1 Evaluation of catalytic performance of *x*Cu/Ni–Al_2_O_3_ catalyst

The catalytic performance of the bimetallic catalyst M/Ni–Al_2_O_3_ (M denotes various metal additives) in the hydrogenation of *N*,*N*-DMA to DMCHA was evaluated in a continuous-flow fixed-bed reactor. Catalyst activity and selectivity were assessed by monitoring the conversion of *N*,*N*-DMA, the selectivity toward the desired product DMCHA, and the formation of the primary by-product *N*-methylcyclohexylamine (*N*-Me-CyH) across different experimental parameters. The catalyst features nickel as the primary active component and incorporates different metal promoters. Five additives (Cu, Ru, Fe, Co, and Sn) with an M/Ni atomic ratio of 0.1 were introduced into the Ni–Al_2_O_3_ precursor, and their catalytic performances were systematically compared under identical reaction conditions. The catalytic performance is shown in Table S2, the catalyst incorporating the Cu additive exhibits the most favorable catalytic performance, achieving a target product selectivity of 94.5% while maintaining high hydrogenation activity, Therefore Cu was ultimately selected as the optimal additive. Therefore, a series of Cu modified Ni–Al_2_O_3_ catalysts with various Cu/Ni ratios (*x*Cu/Ni–Al_2_O_3_) were investigated in the selective hydrogenation of *N*,*N*-dimethylaniline.

As presented in Table 2, when the molar ratio of Cu/Ni increased from 0.05 to 0.5, the conversion rate of *N*,*N*-DMA declined gradually, suggesting that Ni serves as the primary active site. A higher Ni loading enhances the adsorption and activation of H_2_ molecules, thereby generating more active hydrogen species. The exposed Ni sites on the catalyst surface facilitate the attack of hydrogen atoms on the aromatic ring, promoting the cleavage of C–C bonds within the benzene ring.^40^ This process enables effective hydrogen addition to the aromatic ring in *N*,*N*-DMA, leading to the formation of DMCHA and maintaining a high conversion. However, excessive Cu incorporation reduces the availability of Ni active sites, resulting in diminished conversion efficiency. Consequently, by optimizing the Cu loading, specifically at a Cu/Ni molar ratio of 0.1, not only is a high *N*,*N*-DMA conversion rate preserved, but the selectivity toward DMCHA is also enhanced. This improvement is attributable to the moderating effect of Cu on excessive hydrogenation. A side reaction commonly observed with highly active Ni-based catalysts. Under such catalysis, over-hydrogenation may lead to C–N bond cleavage in DMCHA, yielding undesirable by-products (*e.g.*, *N*-Me-CyH, cyclohexanol, and cyclohexane) that compromise product selectivity. The introduction of Cu with appropriate amount helps reduce the d band electrons of Ni, thereby weakening electron occupancy in the antibonding orbitals of the C–N bond in DMCHA,^41^ suppressing its premature cleavage and minimizing side reactions. As a result, the 0.1Cu/Ni–Al_2_O_3_ catalyst demonstrates superior performance in the hydrogenation of *N*,*N*-DMA, achieving both high activity and optimal selectivity.

**Table 2:** Effect of Cu loading on the catalytic performance of the prepared catalysts^a^

Catalyst	*N*,*N*-DMA conversion (%)	DMCHA selectivity (%)	*N-*Me-CyH selectivity (%)
Ni–Al_2_O_3_	89.5	89.1	8.6
0.05Cu/Ni–Al_2_O_3_	89.3	90.4	7.4
0.1Cu/Ni–Al_2_O_3_	89.2	94.5	3.9
0.3Cu/Ni–Al_2_O_3_	81.6	95.6	3.5
0.5Cu/Ni–Al_2_O_3_	77.4	95.9	3.0

a Reaction conditions: 20 mL catalyst, *P* = 5 MPa, *T* = 120 °C, LHSV = 0.2 h^−1^ and H_2_/*N*,*N*-DMA (v/v) = 3500.

On the monometallic Ni-based catalyst, the strong adsorption of Ni toward both the reactant (*N*,*N*-DMA) and protons (H^+^), coupled with the presence of extensive contiguous Ni active sites on the surface, facilitates multipoint adsorption. This configuration promotes over-hydrogenation, manifesting as undesired demethylation or deamination side reactions. Introducing an optimal amount of Cu (*e.g.*, Cu/Ni ratio = 0.1) modulates the electronic structure of Ni: owing to Cu's broader d-band center and its greater energetic separation from the Fermi level relative to Ni, the adsorption strength of Ni toward unsaturated bonds is attenuated.^42^ This electronic effect between Cu and Ni is consistent with the XPS results. Meanwhile, this moderated adsorption energy favors end-on (terminal) adsorption of the reactant, thereby accelerating the initial H atom insertion (*i.e.*, primary hydrogenation) while suppressing flat-lying or multipoint adsorption modes.^43,44^ Consequently, over-hydrogenation is effectively inhibited, and selectivity toward the target product (DMCHA) is maximized. Furthermore, the prominent high-temperature reduction peak β observed in H_2_-TPR for the 0.1Cu/Ni–Al_2_O_3_ catalyst provides direct evidence for the formation of strongly interacting Cu–Ni alloy clusters. Such alloying not only enhances structural stability but also induces electron redistribution at Ni sites *via* ligand effects, lowering the binding energy of DMCHA and facilitating its timely desorption. This mitigates secondary reactions arising from prolonged surface residence, a critical factor in boosting product selectivity.

In contrast, excessive Cu loading (*e.g.*, 0.5Cu/Ni–Al_2_O_3_) further improves low-temperature reducibility, yet catalytic evaluation reveals that surplus Cu species may physically occlude Ni active sites and diminish the density of synergistic Ni–Cu interfacial sites. Although primary hydrogenation activity remains appreciable, the number of sites available for the reactants to undergo specific configuration adsorption decreases, resulting in disordered reaction pathways and decreased conversion rate, reflecting the volcano-type relationship between catalytic structure and activity.

#### 3.2.2 Effects of reaction parameters on hydrogenation of *N*,*N*-DMA

**Fig. 6:**
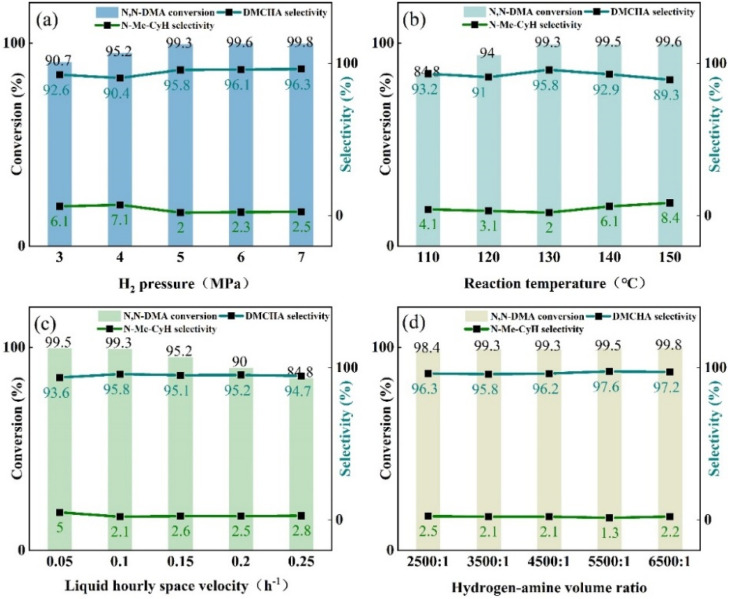
Effects of (a) H_2_ pressure, (b) reaction temperature, (c) LHSV and (d) hydrogen-amine volume ratio on the *N*,*N*-DMA hydrogenation to DMCHA.

The effect of reaction parameters on the hydrogenation of *N*,*N*-DMA under the catalysis of 0.1Cu/Ni–Al_2_O_3_ was systematically evaluated, including hydrogen pressure, reaction temperature, liquid hourly space velocity (LHSV), and hydrogen-amine volume ratio. As illustrated in Fig. 6(a), under the selected conditions, both the conversion of *N*,*N*-DMA and the selectivity toward DMCHA generally increased with rising hydrogen pressure. When the pressure was increased from 3.0 MPa to 7.0 MPa, the reactant conversion improved from 90.7% to 99.8%, while DMCHA selectivity reached a maximum of 96.3%. These results indicate that elevated hydrogen pressure enhances both substrate conversion and product selectivity. However, the selectivity toward the main by-product *N*-Me-CyH was minimized at 5.0 MPa. Further elevating the reaction pressure leads to higher reactant concentrations per unit volume and more extensive interaction with the catalyst's active sites, thereby promoting side reactions and by-product formation. Meanwhile, high pressure requires more powerful equipment and more sophisticated control systems, driven by economic and safety considerations. Consequently, a hydrogen pressure of 5.0 MPa was selected as the optimal condition for subsequent investigations.

Reaction temperature significantly influences catalytic activity. Elevating the reaction temperature enhances the diffusion rate of *N*,*N*-DMA, thereby promoting stronger interaction between the reactant molecules and the metallic Ni sites within the catalyst. This leads to improved conversion of *N*,*N*-DMA and enhanced selectivity toward DMCHA. As illustrated in Fig. 6(b), the effect of temperature on the performance of the 0.1Cu/Ni–Al_2_O_3_ catalyst was investigated by increasing the temperature from 110 °C to 150 °C. The results indicate that the conversion of *N*,*N*-DMA increases progressively with temperature and reaches a maximum of 99.6% at 150 °C. However, the selectivity toward DMCHA exhibits a non-linear trend, it initially increases and then decreases with further temperature elevation, peaking at 95.8% at 130 °C. In contrast, the selectivity toward *N*-Me-CyH exhibits an initial decline followed by an increase, with a minimum observed at 130 °C. These observations suggest that while higher temperatures accelerate the primary reaction, they also promote side reactions that negatively affect catalytic selectivity. Therefore, a reaction temperature of 130 °C is determined to be optimal for subsequent reaction optimization studies.

The feed rate of reactants exerts a significant influence on the outcome of the hydrogenation reaction. Liquid hourly space velocity (LHSV) is a critical parameter reflecting the residence time of reactants on the catalyst surface. The effect of LHSV on the catalytic performance in the hydrogenation of *N*,*N*-DMA was systematically investigated, and with the results illustrated in Fig. 6(c). When the LHSV increased from 0.05 to 0.25 h^−1^, the conversion of *N*,*N*-DMA decreased from 99.5% to 84.8%. Elevated LHSV indicates higher liquid throughput per unit time, thereby reducing the contact duration between reactant molecules and the active sites on the catalyst surface. This shortened interaction time limits the catalyst's ability to fully facilitate the reaction, resulting in lower conversion efficiency. These findings underscore LHSV as a key determinant of hydrogenation conversion rates. However, DMCHA selectivity remained relatively stable across the tested LHSV range, with no significant variation. The highest selectivity toward the desired product DMCHA, along with the lowest formation of the main by-product *N*-Me-CyH, was achieved at an LHSV of 0.1 h^−1^. Therefore, an LHSV of 0.1 h^−1^ is identified as the optimal operational condition for this catalytic system.

The influence of the hydrogen-amine volume ratio was also investigated. Hydrogen functions not only as a reactant in the hydrogenation process but also as a carrier gas, maintaining system pressure stability and ensuring consistent reaction progression. Under selected conditions, the optimal hydrogen-amine volume ratio was experimentally determined. As illustrated in Fig. 6(d), increasing the hydrogen-amine ratio led to higher *N*,*N*-DMA conversion rates, achieving a maximum of 99.8%. This enhancement is attributed to the efficient hydrogenation of *N*,*N*-DMA in a hydrogen-rich environment. However, the selectivity toward the desired product DMCHA peaked at 97.6% when the hydrogen-amine ratio reached 5500 : 1. Further increases in the ratio resulted in decreased DMCHA selectivity, accompanied by increased formation of the by-product *N*-Me-CyH. This trend suggests excessive hydrogenation under such conditions, promoting side reactions and reducing selectivity for DMCHA. Consequently, a hydrogen-amine volume ratio of 5500 : 1 was identified as optimal for achieving high conversion and selectivity simultaneously. Following systematic optimization of various reaction parameters, the substrate conversion rate reached 99.5%, with a corresponding yield of the target product DMCHA of 97.6%. This result represents a notable improvement compared to previous studies,^9^ which reported a maximum yield of 90% under similar conditions in a continuous-flow fixed-bed reactor system.

#### 3.2.3 Stability test of 0.1Cu/Ni–Al_2_O_3_

**Fig. 7:**
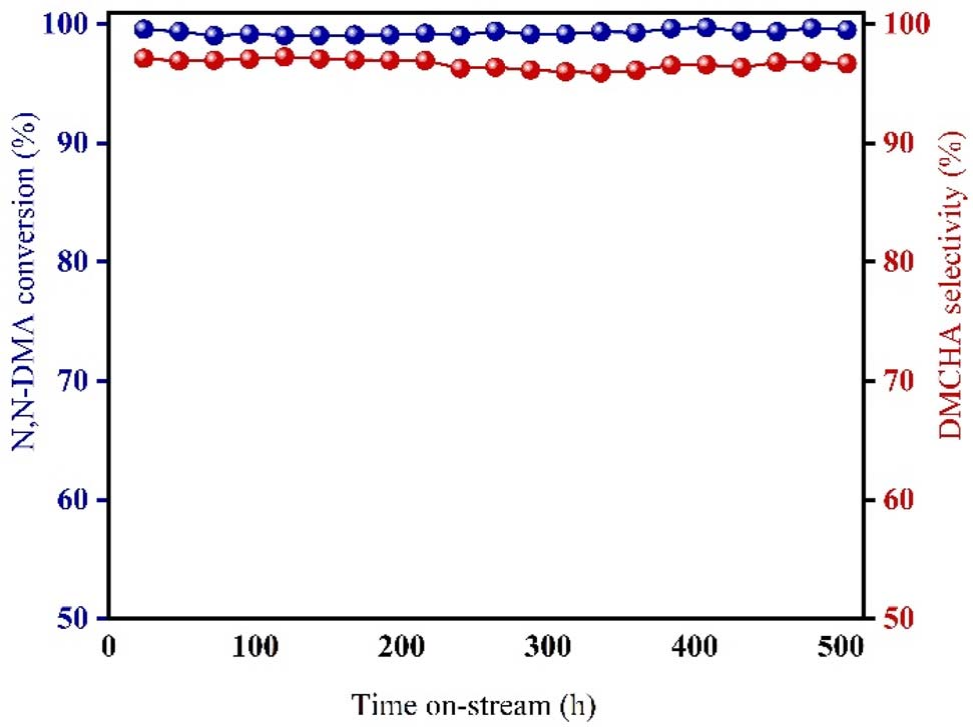
Stability test of 0.1Cu/Ni–Al_2_O_3_.

The 0.1Cu/Ni–Al_2_O_3_ catalyst was evaluated under continuous fixed-bed operation for 500 hours. During this period, the conversion of *N*,*N*-DMA remained consistently above 99.0%, while the selectivity toward DMCHA exceeded 95.0% throughout the test, with the results shown in Fig. 7. Moreover, XPS analysis of the recovered catalyst shows that the binding energies of Ni^0^, Ni^2+^ and Cu^0^ have undergone minor changes, indicating that electron transfer occurred during the catalytic process (Fig. S2). The core catalytically active structure of the catalyst remained stable, and high conversion and high selectivity were maintained throughout the 500-hours continuous reaction. These results demonstrate the catalyst's high catalytic activity, exceptional long-term stability, and strong potential for scalable industrial application.

## 4 Conclusions

In conclusion, the synthesis of DMCHA has been successfully achieved *via* the hydrogenation of *N*,*N*-DMA in a fixed-bed reactor employing a non-noble metal catalytic system. By incorporating Cu as a promoter into the Ni–Al_2_O_3_ precursor *via* an impregnation method, the resulting 0.1Cu/Ni–Al_2_O_3_ catalyst demonstrates excellent catalytic performance and stability, as well as high selectivity toward DMCHA. The well-developed specific surface area and mesoporous structure of the Al_2_O_3_ support facilitate highly dispersed distribution of active metal components. This structural feature promotes more efficient adsorption and activation of H_2_ molecules into reactive hydrogen species, thereby enhancing overall reaction conversion. Furthermore, an electronic interaction between nickel and copper is observed in the *x*Cu/Ni–Al_2_O_3_ catalyst system, which influences the primary hydrogenation pathways and product selectivity. The relative contents of nickel and copper play a critical role in determining catalytic behavior; specifically, at a Cu/Ni molar ratio of 0.1, the catalyst effectively suppresses C–N bond cleavage, minimizes side reactions, and significantly improves selectivity toward the desired product. The effects of reaction parameters-including H_2_ pressure, reaction temperature, LHSV and hydrogen-amine volume ratio on the hydrogenation process were systematically investigated. Ultimately, this study demonstrates a highly selective hydrogenation process for converting *N*,*N*-DMA to DMCHA, underscoring its significant potential for practical application.

## Author contributions

Jingdong Nong: conducting experiments, data curation, and writing original draft. Zhonghua Sun, Weiyou Zhou: writing – review & editing. Zhong Wu: investigation. Junfeng Qian: supervision, project management, and funding acquisition. Qun Chen, Mingyang He: resources, supervision, and funding acquisition.

## Conflicts of interest

There are no conflicts to declare.

## Supplementary Material

RA-016-D6RA01119E-s001

## Data Availability

The data obtained from this research experiment are fully presented in the article and supplementary information (SI). The authors will provide any additional requested data upon reasonable request. Supplementary information: the SI of this research experiment includes physical illustrations of the main experimental equipment (fixed-bed reactor), hydrogenation synthesis schemes, catalyst stability assessment, elemental content table of 0.1Cu/Ni–Al_2_O_3_ catalyst, hydrogenation performance of different additives, gas phase spectra and gas chromatographic spectra of the products, for reference. See DOI: https://doi.org/10.1039/d6ra01119e.
