# Supplementary material for: Efficient selective hydrogenation of N,N-dimethylaniline in a continuous fixed-bed reactor over a Cu/Ni–Al2O3 catalyst
Source: RSC Adv. 2026 Apr 13;16(21):19213–24. doi: 10.1039/d6ra01119e (PMC13075126; doi:10.1039/d6ra01119e)

Supplementary Information (SI) for RSC Advances.
This journal is © The Royal Society of Chemistry 2026

Supporting Information for:

Efficient Selective Hydrogenation of N,N-Dimethylaniline in a Continuous Fixed-bed Reactor over Cu/Ni-Al_2O_3 Catalyst

Jingdong Nong,[a] Zhonghua Sun,[a] Weiyou Zhou,[a] Zhong Wu,[a] Junfeng Qian,[a] Qun Chen,[a] Mingyang He[a]

[a] *Jiangsu Key Laboratory of Advanced Catalytic Materials and Technology, Changzhou University, 213164 Changzhou, China*

Table of Contents

Experimental 3

Characterization of recycled 0.1Cu/Ni-Al_2O_3 catalyst 4

The HR-TEM images for 0.1Cu/Ni-Al_2O_3 catalyst 5

Table S1. The element contents of the 0.1Cu/Ni-Al_2O_3 catalyst analyzed by SEM-EDS 6

Table S2. Effect of metal additives on hydrogenation performance 7

Gas chromatograms of raw materials and products (quantitative analysis) 8

Gas chromatography-mass spectrometry of raw materials and products (qualitative analysis) 9

Experimental

Chemicals and reagents

All the reagents and solvents in the study were analytically pure and were all purchased from Aladdin or Sinopharm Chemical Reagent Co., Ltd. (Shanghai, China) and used as received.

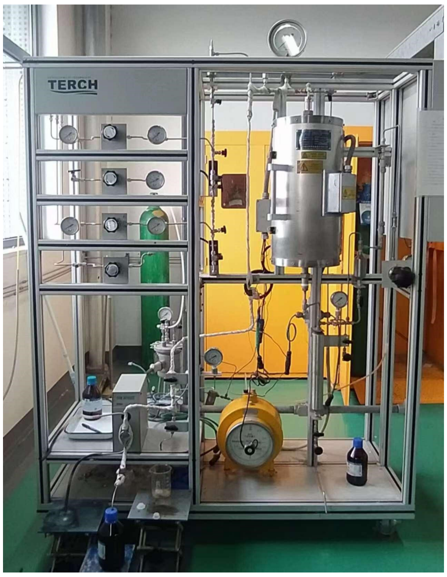

Fig. S1 The photograph of the continuous fixed-bed reactor and the reaction.

Catalytic Hydrogenation of N,N-Dimethylaniline over a Fixed-Bed Reactor

N,N-Dimethylaniline (N,N-DMA) and hydrogen gas were introduced into a fixed-bed reactor at a volumetric feed ratio of 1:5500 (N,N-DMA:H_2). The reaction was conducted under continuous flow conditions at 130 °C and 5 MPa H_2 pressure, using 20 mL of 0.1 wt% Cu/Ni–Al_2O_3 catalyst. After an initial 4-hour stabilization period, steady-state sampling was performed at 2-hour intervals for a total of three consecutive samples. Product composition was determined quantitatively and qualitatively via gas chromatography (GC) and gas chromatography–mass spectrometry (GC–MS).

Characterization of recycled 0.1Cu/Ni-Al_2O_3 catalyst

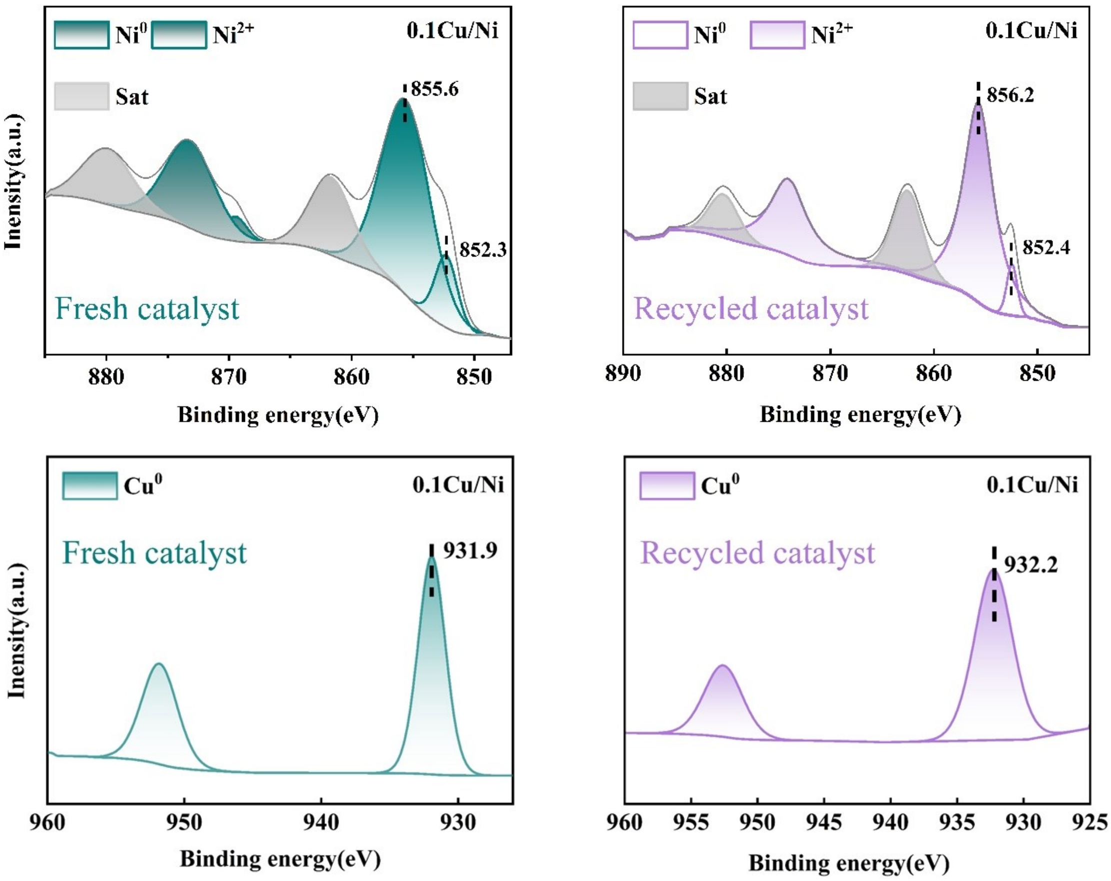

Fig. S2 XPS spectra for fresh and recycled 0.1Cu/Ni-Al_2O_3 catalyst.

The HR-TEM analysis for 0.1Cu/Ni-Al_2O_3 catalyst

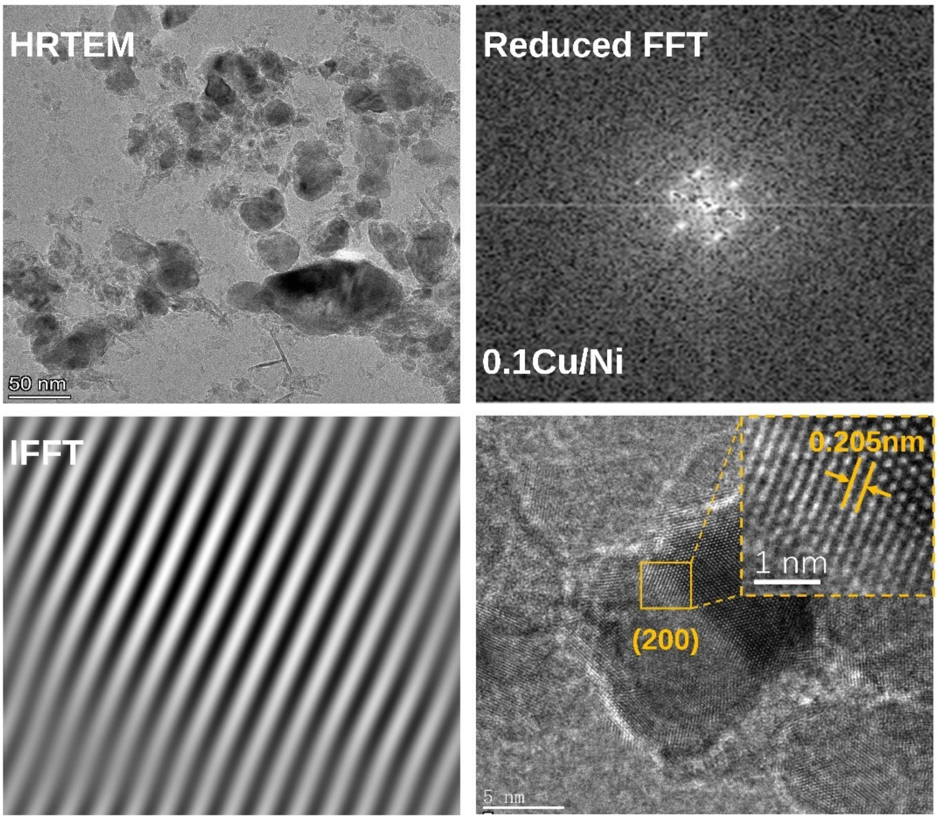

Fig. S3 The HR-TEM analysis for 0.1Cu/Ni-Al_2O_3 catalyst.

The data in Table S1 were obtained through scanning electron microscopy and X-ray energy spectrum analysis for the 0.1Cu/Ni-Al_2O_3 catalyst with the best catalytic performance. The atomic ratio of Cu to Ni in this catalyst was close to the theoretical value (Cu/Ni = 0.1)

Table S1. The element contents of the 0.1Cu/Ni-Al_2O_3 catalyst analyzed by SEM-EDS

Element	Line Type	Weight %	Weight Sigma %	Atomic %
O	K series	40.55	1.06	57.76
Al	K series	42.10	1.05	35.56
Ni	K series	15.72	1.05	6.10
Cu	L seies	1.62	1.93	0.58
Total		100		100

The data in Table S2 is used to investigate the effects of five different metal additives (Cu, Ru, Fe, Co, and Sn) on the hydrogenation performance of the catalyst Ni-Al_2O_3 for N,N-DMA. During the investigation, the loading amount of the additives and the reaction conditions were kept consistent. The results include the conversion rate of the raw material N,N-DMA, the selectivity of the product DMCHA, and the selectivity of the main by-product N-Me-CyH.

Table S2. Effect of metal additives on hydrogenation performance

Metal additives	N,N-DMA Conversion (%)	DMCHA Selectivity (%)	N-Me-CyH Selectivity (%)
--	89.5	89.1	8.6
Cu	89.4	94.5	3.9
Ru	89.4	86.1	7.7
Fe	89.3	91.2	6.6
Co	89.2	83.0	8.5
Sn	89.5	90.7	7.1

Reaction conditions: 20 ml catalyst, P = 5 MPa, T = 120 °C, LHSV = 0.2 h^{-1} and H_2 / N,N-DMA (v/v) = 3500, X/Ni molar ratio = 0.1

Gas chromatograms of raw materials and products (quantitative analysis)

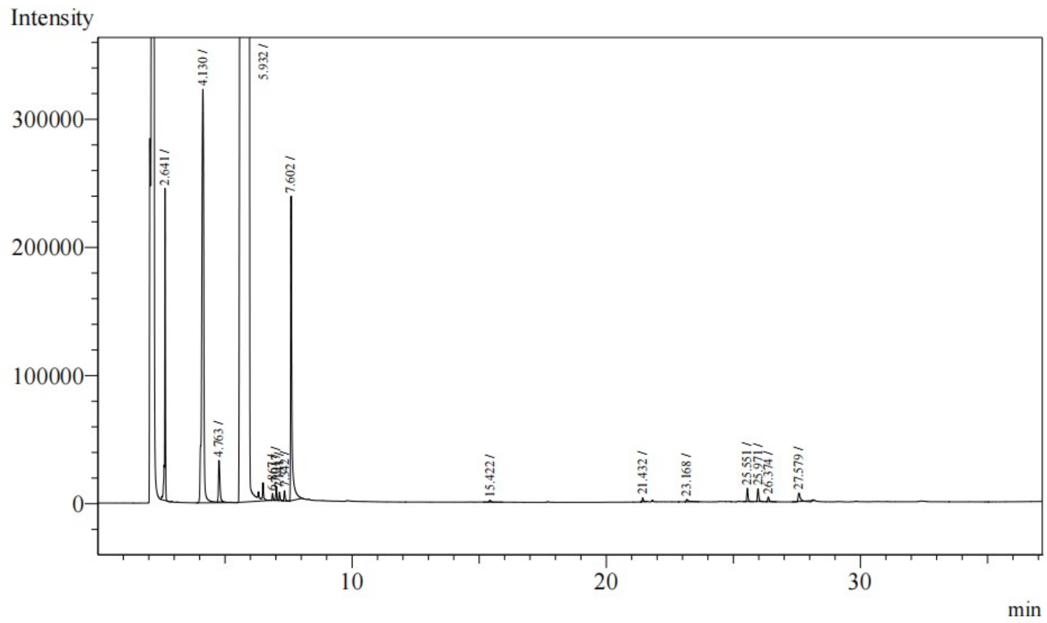

Peak#	Ret.Time	Area	Height	Conc.	Unit	Mark	ID#	Cmpd Name
1	2.641	455069	223130	0.317				
2	4.130	1798767	321068	1.252		S		
3	4.763	129837	32444	0.090		T		
4	5.932	140284498	12006935	97.630				
5	6.867	14126	5114	0.010				
6	7.017	28452	11000	0.020				
7	7.141	17886	6529	0.012				
8	7.342	23576	7942	0.016				
9	7.602	792860	231260	0.552				
10	15.422	7919	1537	0.006				
11	21.432	8413	3268	0.006				
12	23.168	14581	1826	0.010				
13	25.551	33526	10296	0.023				
14	25.971	36277	9904	0.025				
15	26.374	14825	3639	0.010				
16	27.579	28793	6574	0.020				
**Total**		143689405	12882466					

Product main ingredient list

Peak	1	2	3	4	9
Component	Cyclohexane	N-methylcyclohexylamine	Cyclohexanol	N,N-dimethylcyclohexylamine	N,N-dimethylaniline
Content (%)	0.3	1.25	0.1	97.6	0.5

Gas chromatography-mass spectrometry of raw materials and products (qualitative analysis)

Product Gas chromatography mass spectrum overview

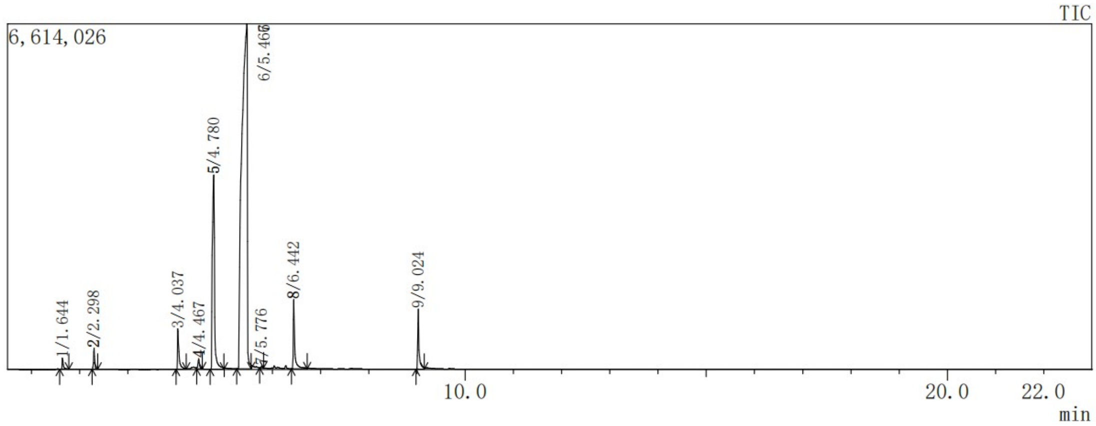

Component name: N,N-dimethylcyclohexylamine

Molecular formula: C8H17N, CAS number: 98-94-2, Molecular weight: 127, Retention index: 929

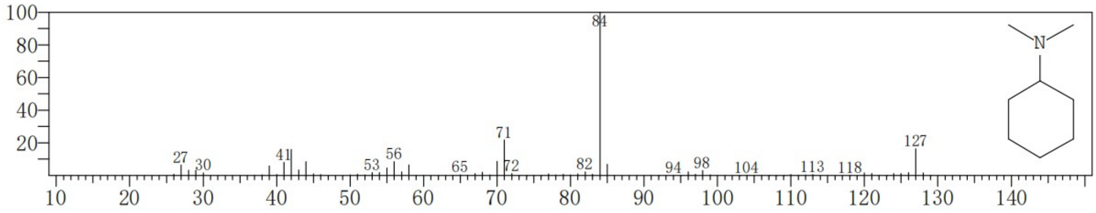

Component name: N-methylcyclohexylamine

Molecular formula: C7H15N, CAS number: 100-60-7, Molecular weight: 113, Retention index: 979

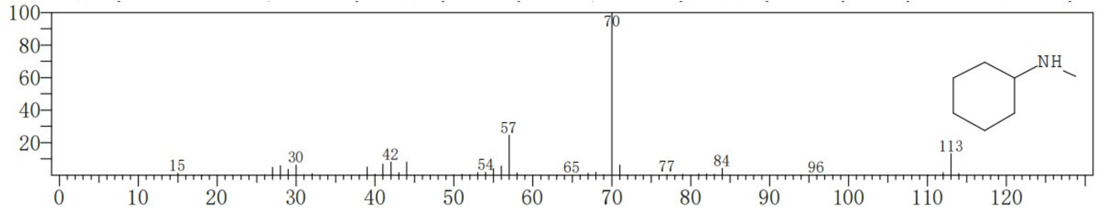

Component name: Cyclohexanol

Molecular formula: C6H12O, CAS number: 108-93-0, Molecular weight: 100, Retention index: 908

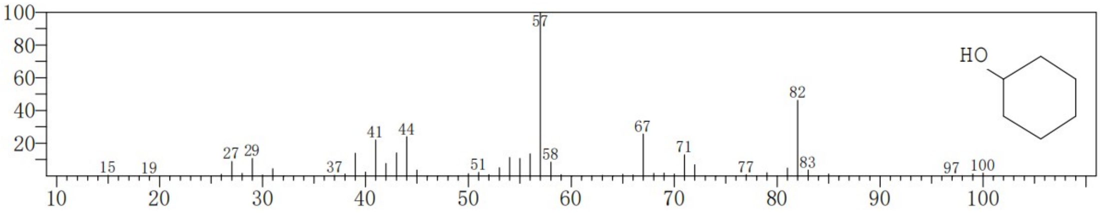

Component name: Cyclohexane

Molecular formula: C6H12, CAS number: 110-82-7, Molecular weight: 84, Retention index: 719

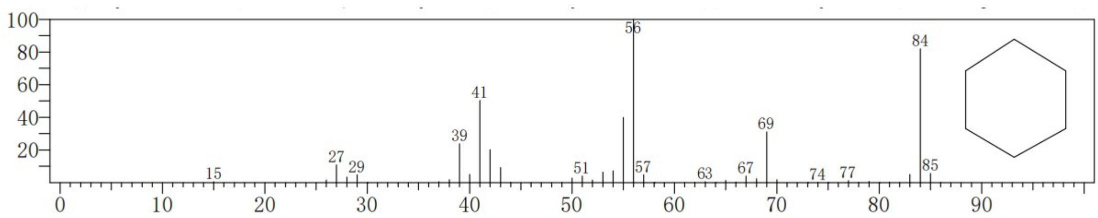

Component name: N,N-dimethylaniline

Molecular formula: C8H11N, CAS number: 121-69-7, Molecular weight: 121, Retention index: 942

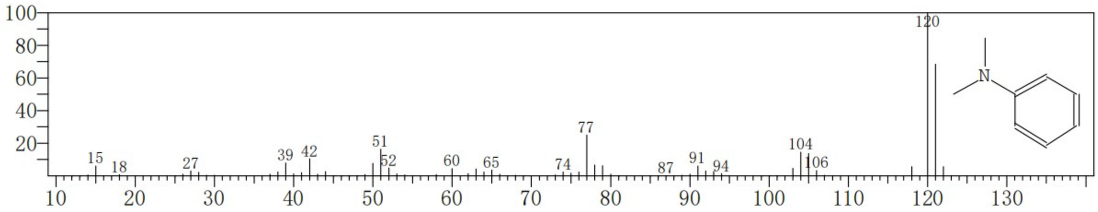

Supplement: RA-016-D6RA01119E-s001 [file RA-016-D6RA01119E-s001.pdf]
